# Morphology-controlled synthesis of CuCo_2_S_4_ as a high-efficiency counter electrode *via* a precursor-directed strategy for quantum dot-sensitized solar cells (QDSSCs)

**DOI:** 10.1039/d5ra06421j

**Published:** 2025-10-27

**Authors:** Qiu Zhang, Yuekun Zhang, Chunxiao Zhang, Xiuyan Jiang, Xuemei Fu

**Affiliations:** a School of Chemical Engineering, Shandong Institute of Petroleum and Chemical Technology Dongying 257061 China 2023042@sdipct.edu.cn; b Shandong Key Laboratory of Green Electricity & Hydrogen Science and Technology, Shandong Institute of Petroleum and Chemical Technology Dongying 257061 China; c Dongying Key Laboratory of New Energy Materials and Devices, School of Chemical Engineering, Shandong Institute of Petroleum and Chemical Technology Dongying 257061 China

## Abstract

Quantum dot-sensitized solar cells (QDSSCs) have emerged as promising photovoltaic devices, in which the counter electrode (CE) plays a crucial role in the catalytic reduction of S_n_^2−^ and charge transfer. Based on a precursor-directed strategy, this study reports a simple and cost-effective solvothermal method for the synthesis of morphology-controlled CuCo_2_S_4_ nanomaterials without templates and structure-directing agents, including flower-like (f-CuCo_2_S_4_), nanosheet-like (n-CuCo_2_S_4_), nanoparticle-like (p-CuCo_2_S_4_), and microsphere-like (m-CuCo_2_S_4_) structures. The effects of CuCo_2_S_4_ CEs with different morphologies on the photovoltaic performance of QDSSCs were also systematically investigated. Among them, the f-CuCo_2_S_4_ CE exhibited the highest specific surface area and the best catalytic performance, resulting in a power conversion efficiency (PCE) of 7.42% for QDSSCs, which is 55% higher than that of materials with other morphologies. Electrochemical analysis confirmed that it delivered the lowest charge transfer resistance (*R*_ct_ = 0.076 Ω) and the highest electrocatalytic activity. This work highlights the importance of morphology control for optimizing the performance of CEs for efficient QDSSCs.

## Abstract 2

Different morphologies of CuCo_2_S_4_-CEs were synthesized through a precursor-directed strategy; the QDSSCs based on f-CuCo_2_S_4_-CEs achieve a PCE of 7.42%.

## Graphical abstract

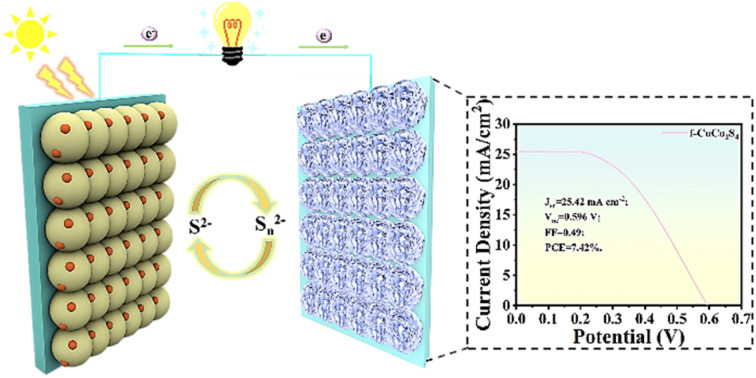

## Introduction

With the continuous development of human society, the demand for energy is also increasing.^1,2^ However, the extensive exploitation and use of traditional fossil energy has caused serious energy crisis and environmental pollution problems.^3^ Therefore, it is particularly important to adjust the energy structure. Among many renewable energy sources, solar energy is widely favored because of its green environmental protection, abundant reserves and low price.^4^ Furthermore, among the many basic forms of utilizing solar energy, quantum dot-sensitized solar cells (QDSSCs), as one of the representatives of the third generation of emerging photovoltaic cells, have attracted widespread attention due to the fascinating properties of quantum dots used for light collection, such as adjustable band gap, high molar extinction coefficient, large intrinsic dipole moment and dielectric confinement effect.^5,6^ Meanwhile, the potential of multi-exciton generation can make its theoretical conversion efficiency break the Shockley–Queisser limit (33%) and reach 44%,^7,8^ making it one of the promising candidates for the development of green renewable energy.^9^

Classic QDSSCs have a sandwich structure similar to dye-sensitized solar cells (DSSCs),^10,11^ mainly including four basic elements: quantum dot sensitizer, photoanode, electrolyte and counter electrode (CE).^12^ Among them, QDs are used to absorb sunlight to generate electron–hole pairs. The photoanode not only acts as an electron transfer bridge, but also bears the role of loading QDs.^13^ The role of the electrolyte is to achieve the regeneration of the sensitizer.^14^ As an indispensable component of QDSSCs, the CE plays a pivotal role. Like an efficient catalyst, it is responsible for collecting and transferring electrons from the external circuit and catalyzing the reduction of oxidized electrolytes, ensuring that the chemical reactions inside the cell can proceed smoothly.^15,16^ Thus, each individual component significantly influences the diverse parameters of the cell. Throughout the existing research, the focus is mainly on the photoanode and electrolyte, while the reports on the CE are relatively few.

As a commonly used counter electrode material, the QDSSCs assembled with traditional brass foil-based Cu_2_S-CE exhibit relatively higher power conversion efficiency (PCE).^17^ However, the polysulfide electrolyte often corrodes the brass substrate continuously, resulting in Cu_2_S film falling off.^18^ Additionally, its relatively low specific surface area limits the rapid catalytic reduction reaction, then resulting in continuous decline in the mechanical stability and optoelectronic performance of the QDSSCs. Although the catalytic performance and stability of Cu_*x*_S-based counter electrodes can be enhanced through optimization of the preparation process, the performance of CE materials composed of single metal sulfides is usually limited by the unsatisfactory properties of the single component,^19,20^ which makes the performance of QDSSCs devices unable to be further improved. Compared with single metal sulfides, bimetallic transition metal sulfides, benefiting from the synergistic effect among different types of mixed-valence metal ions, can generate abundant redox active sites, providing the required low activation energy for electron transfer, thereby significantly enhancing the overall catalytic performance of the material.^21,22^

In recent years, owing to their high intrinsic electrical conductivity, excellent structural stability, and remarkable synergistic catalytic performance, transition metal sulfides AB_2_S_4_ with spinel structure, such as CuCo_2_S_4_, NiCo_2_S_4_, MnCo_2_S_4_, CuFe_2_S_4_, *etc.*, have been widely applied in the fields of supercapacitors, lithium-ion batteries, electrochemical sensing, oxygen evolution reactions, and photovoltaic cells.^23–27^ Among them, CuCo_2_S_4_ has the advantages of abundant reserves and non-toxicity.^28^ Moreover, copper-based materials are of low cost and possess excellent electrical conductivity, while cobalt-based materials exhibit excellent catalytic activity similar to that of noble metals.^29^ Therefore, in recent years, as a typical representative of spinel sulfides, CuCo_2_S_4_ has been extensively studied as the CE material for DSSCs.^30,31^ Sambandam Anandan *et al.* prepared CuCo_2_S_4_ nanosheets by ultrasonic synthesis method under low-temperature environmental conditions, and applied them to DSSC as CE catalyst, obtaining a PCE of 10.21%.^32^ Bakhytzhan Baptayev used the nanoflower-like CuCo_2_S_4_ prepared by the solvothermal method as the CE of DSSC, achieving a PCE of 7.56%.^33^ However, to the best of our knowledge, there are still few studies on applying CuCo_2_S_4_ as a CE in QDSSCs. Thus, the preparation of spinel Cu and Co metal sulfides into high-efficiency electrode in QDSSCs still has great development space.

Furthermore, the catalytic performance of CEs is closely related to the morphology of the material.^34^ The design of specific morphologies can not only increase the exposure of the active surface area and the number of catalytic sites, but also facilitate efficient electron transfer and electrolyte diffusion.^11,35^ As a result, reducing the charge transfer resistance and optimizing the redox kinetics. Nevertheless, in QDSSCs, there are still relatively few studies systematically exploring the influence of the morphology on the performance of CEs. Although a few studies have reported the synthesis methods of CuCo_2_S_4_ with definite morphology, template agents or structure-directing agents are usually required during the synthesis process.^36,37^ This makes the preparation method complicated, to a certain extent, restricts the widespread application of CuCo_2_S_4_. Thus, this study demonstrates a straightforward precursor-directed strategy for preparing CuCo_2_S_4_-CEs with diverse morphologies, and it is discovered that the nanoflower-like CuCo_2_S_4_ (f-CuCo_2_S_4_) has a large specific surface area and a three-dimensional porous structure, which can amplify the synergistic effect between Cu and Co ions, and exhibit excellent electrocatalytic activity towards polysulfide electrolytes. The QDSSCs based on the f-CuCo_2_S_4_-CE can achieve a PCE of 7.42% with *J*_sc_ = 25.42 mA cm^−2^, *V*_oc_ = 0.596 V, (FF = 0.49), which is 24%, 39%, and 55% higher than that of the CuCo_2_S_4_-CE in the form of nanosheets (n-CuCo_2_S_4_), nanoparticles (p-CuCo_2_S_4_), and microspheres (m-CuCo_2_S_4_) respectively.

## Experimental

### Synthesis of flower-like CuCo_2_S_4_ and other CE materials

**Fig. 1:**
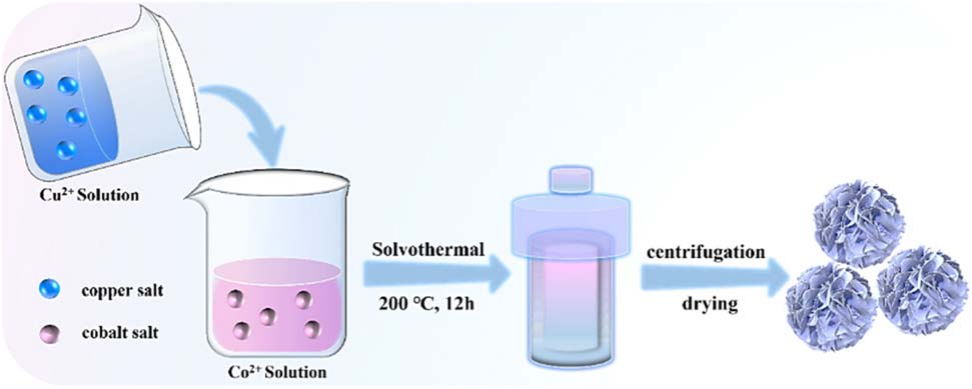
Schematic diagram of the synthesis of f-CuCo_2_S_4_.

The flower-like CuCo_2_S_4_ was prepared by a simple solvothermal reaction method as shown in Fig. 1. Briefly, 0.5 mmol of CuCl_2_·2H_2_O was added to 60 mL of ethylene glycol solution and stirred until it was dissolved. Then, 1 mmol of CoCl_2_·6H_2_O and 3 mmol of thiourea were added to the above solution successively. After continuous stirring for 30 min, the mixed solution was transferred to a 100 mL autoclave and reacted at 200 °C for 12 h, and the heating rate was 2 °C min^−1^. After the reaction cooled down to room temperature, the obtained precipitate was washed three times with deionized water and ethanol respectively. Then, it was transferred into an oven at 60 °C and dried for 10 h, thus the flower-like CuCo_2_S_4_ (f-CuCo_2_S_4_) could be obtained. The detailed synthesis methods of CuCo_2_S_4_ materials with particle-like (p-CuCo_2_S_4_), microsphere-like (m-CuCo_2_S_4_), nanosheet-like (n-CuCo_2_S_4_) morphologies are presented in the SI.

### Preparation of different CE films

Firstly, the commercial FTO (SnO_2_: F) was cleaned with a cleansing solution for 15 minutes. Then, the above-mentioned FTO was ultrasonically cleaned in acetone, deionized water, and ethanol for 30 minutes respectively, and then soaked in ethanol for later use. Next, weigh 0.2 g of the prepared CuCo_2_S_4_ materials with different morphologies (f-CuCo_2_S_4_, p-CuCo_2_S_4_, m-CuCo_2_S_4_, n-CuCo_2_S_4_) respectively, grind them evenly and pour them into an agate mortar. Then, add 0.02 g of ethyl cellulose, 0.8 g of terpineol and 10 mL of ethanol into the mortar. After mixing evenly, keep grinding until the ethanol is completely volatilized, and then different thick CE slurries can be obtained. Meanwhile, different slurries were coated onto the pre-prepared FTO using screen printing technology. After leaving them to stand for 10 min, the samples were placed on a heating plate at 80 °C for drying. Subsequently, they were transferred into a muffle furnace and calcined at 400 °C for 30 min to enhance the adhesion between the CE film and the FTO, the heating rate was 1 °C min^−1^. After cooling down to room temperature, CE films with different morphologies (f-CuCo_2_S_4_, p-CuCo_2_S_4_, m-CuCo_2_S_4_, n-CuCo_2_S_4_) could be obtained.

### Construction of FTO/TiO_2_/CdS/CdSe/ZnS photoanode

2.5 g commercial TiO_2_ (P25) and 15 g terpineol were put into a cleaned ball-milling jar containing agate grinding balls. After ball-milling at room temperature for 12 h, a viscous TiO_2_ slurry was obtained. Then, dropped the obtained TiO_2_ slurry onto the FTO with a fixed area, and used an appropriate scraper to evenly coat the slurry. Additionally, it was placed on a heating plate at 70 °C for drying and transferred into a muffle furnace, annealed at 450 °C for 40 min (the heating rate is 5 °C min^−1^). After cooling down to room temperature, taken it out to receive FTO/TiO_2_.

In addition, 5 mmol of Cd(NO_3_)_2_·4H_2_O was prepared into a 0.1 M methanol solution as a Cd^2+^ source, and 5 mmol of Na_2_S·9H_2_O was dissolved in a mixed solution of methanol and DI (volume ratio 1 : 1) to obtain a 0.1 M S^2−^ source. The calcined photoanode was vertically immersed in the 0.1 M Cd^2+^ source for 2 min, then rinsed with methanol and dried with N_2_. Subsequently, the above film was vertically placed in a 0.1 M S^2−^ source and immersed for 2 min, then rinsed with DI and dried with N_2_. This process is called a successive ion-layer adsorption and reaction (SILAR) cycle. After 6 cycles, FTO/TiO_2_/CdS can be prepared. CdSe-QDs were obtained by chemical bath deposition (CBD). A certain amount of CdSO_4_·8/3H_2_O and N(CH_2_COONa)_3_ (NTA) was weighed to prepare 0.03 M CdSO_4_·8/3H_2_O solution and 0.2 M NTA solution, respectively. According to the volume ratio of 1 : 1 : 1, the prepared Na_2_SeSO_3_ (the preparation method is presented in the SI), CdSO_4_·8/3H_2_O and NTA solution were mixed evenly and stirred continuously at room temperature for 20 min. Subsequently, the prepared FTO/TiO_2_/CdS photoanode was vertically immersed in the above mixed solution and allowed to stand for deposition in a dark environment at 5 °C for 6 h. After being taken out, it was fully rinsed with DI and dried with N_2_ to obtain FTO/TiO_2_/CdS/CdSe. Finally, the SILAR method was used to deposit the ZnS passivation layer. In short, the FTO/TiO_2_/CdS/CdSe photoanode was vertically immersed in the prepared 0.1 M Zn(CH_3_COO)_2_·2H_2_O solution for 2 min. After the electrode was taken out, it was rinsed with DI and dried with N_2_. Then the above electrode was vertically placed in 0.1 M Na_2_S·9H_2_O and immersed for 2 min. When the deposition was completed, the surface residue was fully rinsed with DI and dried with N_2_. After alternating for 3 cycles, the FTO/TiO_2_/CdS/CdSe/ZnS photoanode was received.

### Assembly of QDSSCs

2 M Na_2_S·9H_2_O, 2 M sulfur powder and 0.2 M KCl were stirred continuously for 30 min in a water and methanol solvent with a volume ratio of 7 : 3, and then ultrasonically dissolved for 1 h. After complete dissolution, an orange-yellow polysulfide electrolyte was obtained.

Next, the FTO/TiO_2_/CdS/CdSe/ZnS photoanode and various CEs were fixed together face to face using a dovetail clamp. The prepared electrolyte (40 μL) was injected into the gap between the two electrodes using a pipette, then a sandwich-type QDSSCs was constructed. The symmetric cell was used to test the electrochemical performance of the counter electrode. Except for replacing the same two counter electrodes, the rest of the assembly process was consistent with the assembly of QDSSCs. The effective active area of the QDSSC was designed to be 0.12 cm^2^.

## Results and discussion

**Fig. 2:**
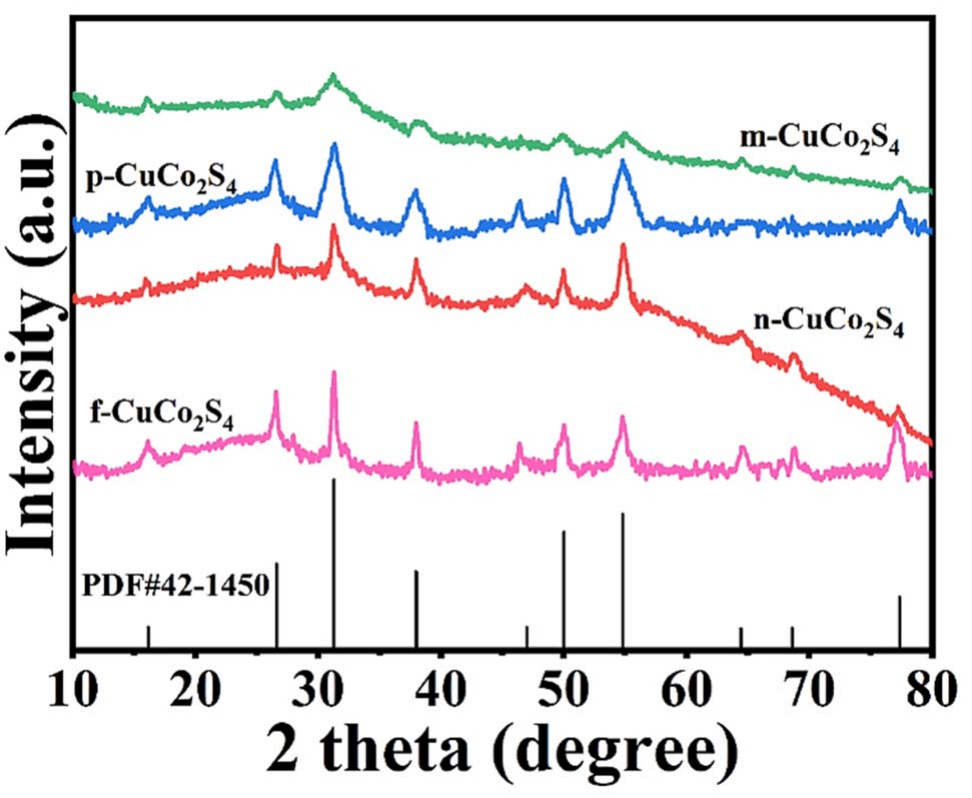
XRD patterns of CuCo_2_S_4_ with different morphologies.

X-ray diffractometer (XRD) was used to characterize the composition and structure of different samples. It can be clearly observed from Fig. 2 that the diffraction peak signals exhibited by the prepared samples all correspond to the standard spectrum of the carrollite structure phase CuCo_2_S_4_ (JCPDS No. 42-1450).^38^ The diffraction peaks at 2*θ* = 16.12°, 26.58°, 31.30°, 37.96°, 49.99°, 54.81° and 77.33° correspond to the (111), (022), (113), (004), (115), (044) and (137) crystal planes of CuCo_2_S_4_, respectively. The above analysis shows that CuCo_2_S_4_ was successfully prepared. In addition, it can be seen from the figure that CuCo_2_S_4_ synthesized from different metal precursors exhibits different degrees of crystallinity. Among all the samples, the flower-like CuCo_2_S_4_ exhibits the highest crystallinity, while the microsphere CuCo_2_S_4_ has the lowest crystallinity, which we speculate can be attributed to the difference in coordination strength of different anions.

**Fig. 3:**
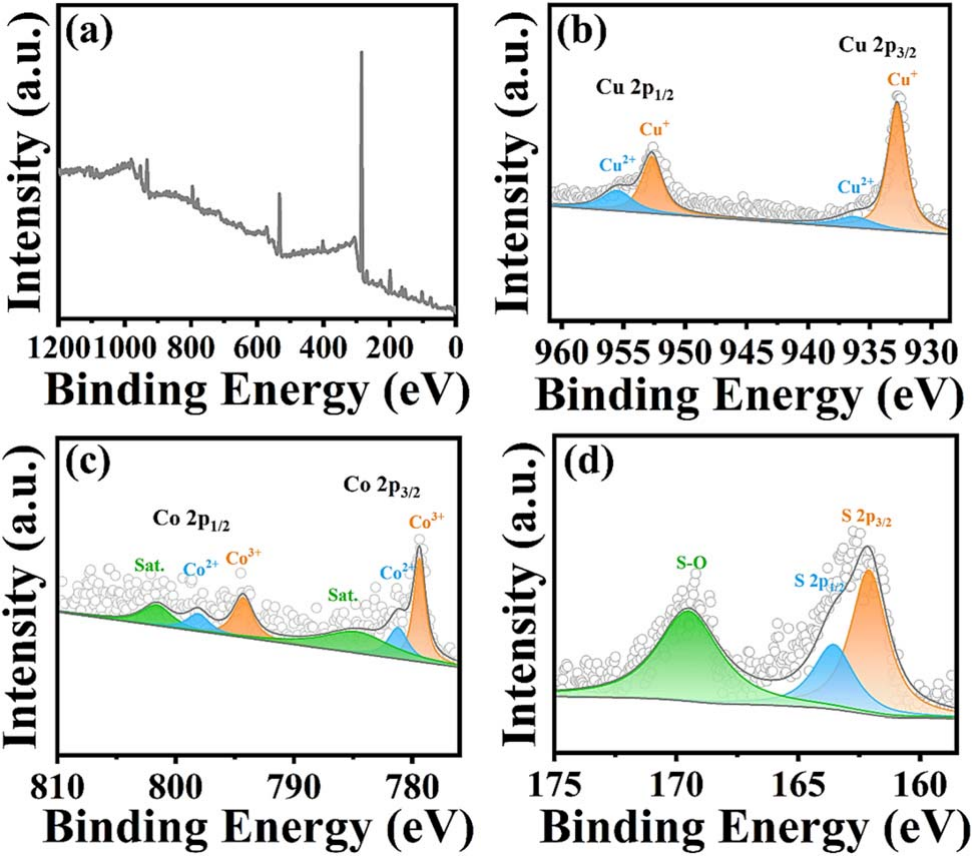
(a) XPS survey spectrum; (b) Cu 2p; (c) Co 2p; (d) S 2p high resolution XPS spectra of flower-like CuCo_2_S_4_.

To better prove the elemental composition and chemical state of the sample, X-ray photoelectron spectroscopy (XPS) was further used to measure the flower-like CuCo_2_S_4_ sample. The XPS survey spectrum presented in Fig. 3(a) shows the presence of signal peaks of three elements (Cu, Co and S) in the sample, further proving the successful preparation of the sample. Additionally, Fig. 3(b) is a high-resolution XPS spectrum of Cu 2p. The binding energy at 932.8 eV and 952.7 eV correspond to the signal peaks of Cu 2p_3/2_ and Cu 2p_1/2_, respectively, confirming the presence of Cu^+^ in the CuCo_2_S_4_ sample.^39^ There are two weak signal peaks at 935.8 eV and 955.5 eV, indicating the presence of Cu^2+^ in the sample. Fig. 3(c) depicts the high-resolution XPS spectrum of Co 2p. The peak in the binding energy range of 779–782 eV points to Co 2p_3/2_, while the characteristic peak signal in the binding energy range of 793–798 eV points to Co 2p_1/2_. Simultaneously, the peaks at 779.1 eV and 794.2 eV binding energy correspond to the characteristic peaks of Co^3+^, the peak signals at 781.2 eV and 797.8 eV binding energy indicate the presence of Co^2+^, and the signal peaks at 784.8 eV and 802.9 eV are usually due to the satellite peaks of Co^2+^. Fig. 3(d) shows the XPS spectrum of S 2p. The S 2p signal peak of the CuCo_2_S_4_ sample can be further deconvoluted into two main peaks (S 2p_3/2_, S 2p_1/2_) at 162.1 eV and 163.5 eV. Furthermore, the low-intensity broad peak near 169.3 eV binding energy may be due to the partial oxidation of the sample surface to form S–O bonds. The above test results are consistent with the XRD results, further proving the successful synthesis of the CuCo_2_S_4_ sample.

**Fig. 4:**
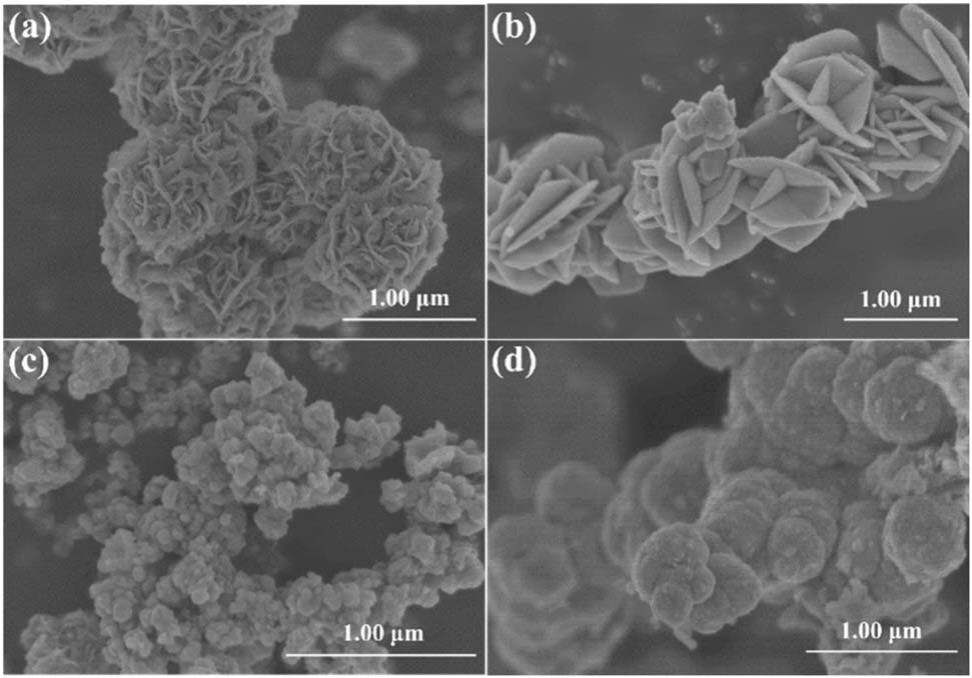
SEM images of (a) flower-like; (b) nanosheet-like; (c) nanoparticle-like; (d) microsphere-like CuCo_2_S_4_.

**Fig. 5:**
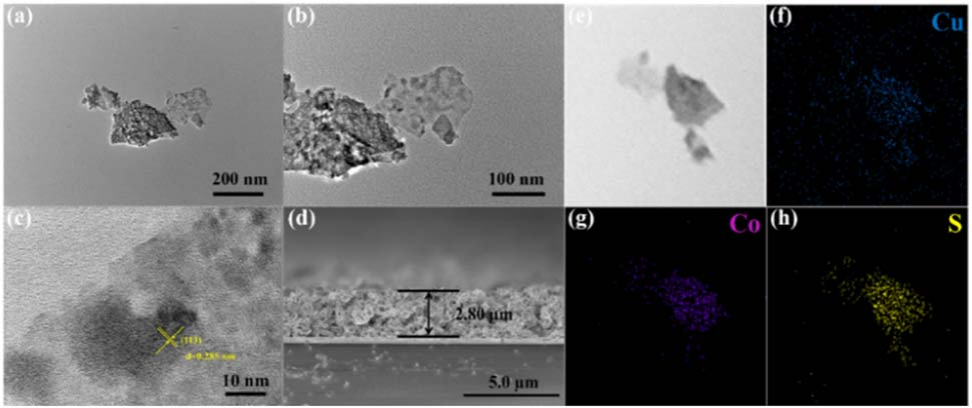
(a and b) TEM images; (c) HRTEM images; (d) SEM cross-sectional view; (e–h) EDS element distribution images of flower-like CuCo_2_S_4_.

The microstructure and morphology of the CuCo_2_S_4_ samples were further characterized using SEM and TEM. As shown in Fig. 4(a), the CuCo_2_S_4_ synthesized using metal chlorides (CuCl_2_·2H_2_O and CoCl_2_·6H_2_O) as precursors exhibited a flower-like nanostructure with a diameter of approximately 1.2 μm. Simultaneously, Fig. 4(b)–(d) respectively show the nanosheet-like CuCo_2_S_4_ (from Cu(NO_3_)_2_·3H_2_O and Co(NO_3_)_2_·6H_2_O), loosely aggregated nanoparticle-like CuCo_2_S_4_ (from CuSO_4_·5H_2_O and CoSO_4_·7H_2_O), and microsphere-like CuCo_2_S_4_ (from Cu(CO_2_CH_3_)_2_·*x*H_2_O and Co(CO_2_CH_3_)_2_·4H_2_O). Obviously, compared with the other morphologies, the flower-like CuCo_2_S_4_ with its open, porous three-dimensional structure is more favorable for increasing the specific surface area of the material, thereby enhancing the number of active catalytic sites and promoting electrolyte infiltration. This indicates that the microscopic morphology of CuCo_2_S_4_ is influenced by the type of metal precursor, and that precise morphological control can be achieved by selecting different precursors, ultimately leading to improved counter electrode performance. Fig. 5(a)–(c) clearly depicts the TEM and high-resolution TEM (HRTEM) images of nanoflower CuCo_2_S_4_. As shown in Fig. 5(a) and (b), the interior of the nanoflower CuCo_2_S_4_ presents an open porous structure, which is consistent with the SEM results. The HRTEM image shown in Fig. 5(c) observed lattice fringes with a lattice spacing of 0.285 nm, corresponding to the (113) crystal plane of the nanoflower CuCo_2_S_4_. Additionally, Fig. 5(d) shows the SEM cross-sectional view of the nanoflower CuCo_2_S_4_ film obtained by screen printing technology. It can be clearly seen that the morphology of the film is relatively dense and the thickness is about 2.80 μm. Furthermore, the film is in close contact with the FTO substrate, which can reduce the series resistance (*R*_s_) of the cell, thereby improving the photovoltaic performance of the QDSSCs.

Elemental mapping scanning and EDS were used to further analyze the elemental composition of the CuCo_2_S_4_ material. The EDS mapping scan results of the CuCo_2_S_4_ are depicted in Fig. 5(e)–(h), and the Cu, Co and S elements are evenly distributed in the sample. Besides, the signal peaks of the EDS spectrum (Fig. S3) indicate the presence of Cu, Co and S elements in all the samples, and each of the elemental analysis report shows that the atomic ratio of Cu, Co and S is close to its stoichiometric ratio. This corresponds to the EDS mapping and further proves the successful synthesis of the CuCo_2_S_4_.

**Fig. 6:**
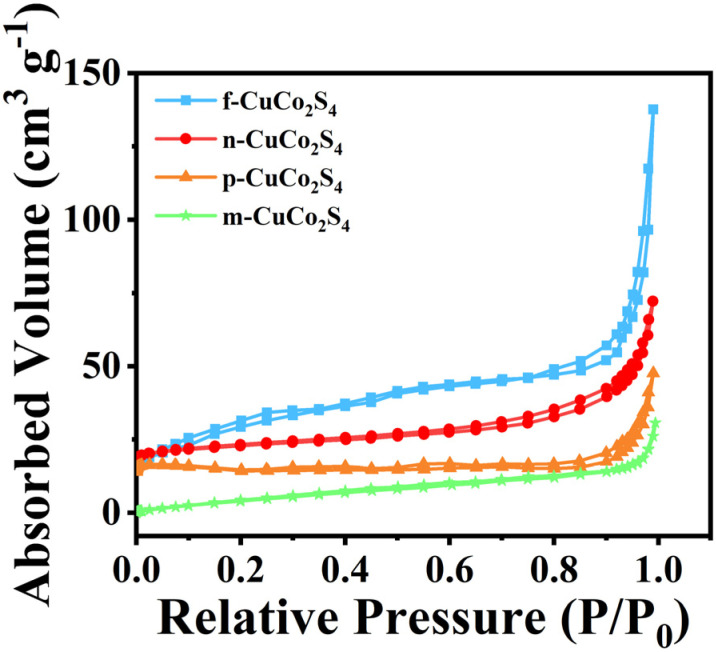
N_2_ adsorption–desorption isotherms of f-CuCo_2_S_4_, n-CuCo_2_S_4_, p-CuCo_2_S_4_, m-CuCo_2_S_4_.

Analyzing the specific surface area and pore structure of the synthesized materials is of great significance for evaluating their performance. As revealed in Fig. 6, the specific surface area and pore structure of the materials were characterized by N_2_ adsorption–desorption isotherms. Apparently, the CuCo_2_S_4_ materials with different morphologies all showed IV-type adsorption isotherms accompanied by H_3_-type hysteresis loops, confirming the existence of mesoporous structures in the materials. The rich mesoporous structure can significantly optimize the catalytic process of the counter electrode, promote the penetration of the electrolyte, and thus improve the overall performance of QDSSCs. The detailed BET data obtained are summarized in Table S1. It can be seen that compared with CuCo_2_S_4_ materials with other morphologies, f-CuCo_2_S_4_ has the largest specific surface area (*S*_BET_ = 44.86 m^2^ g^−1^). In the catalytic reduction process, the large specific surface area can provide more catalytic active sites. Therefore, QDSSCs composed of f-CuCo_2_S_4_ counter electrodes exhibit excellent photovoltaic performance.

**Fig. 7:**
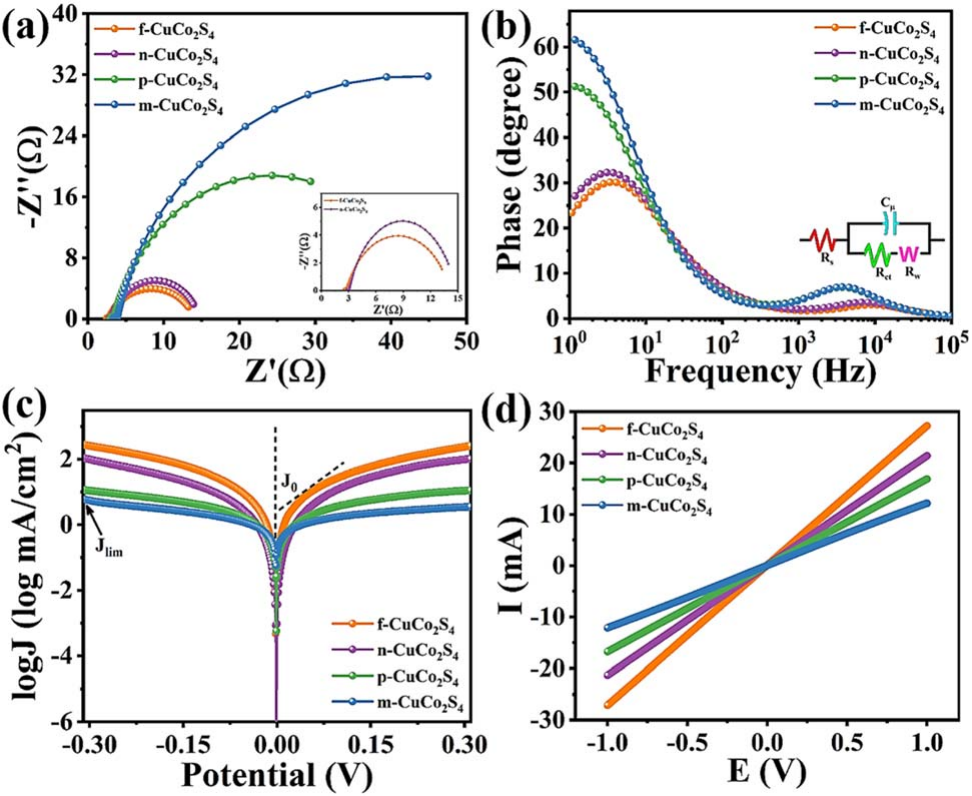
(a) Nyquist diagrams (illustration is partial enlargement of Nyquist plots); (b) corresponding Bode phase diagrams (illustration is equivalent circuit diagram); (c) Tafel polarization curves of symmetric cells based on various CuCo_2_S_4_ CEs; (d) LSV plots for different CEs.

To further explore the electron transfer ability and electrocatalytic activity of different CEs, EIS tests were performed on different CE materials using a symmetrical cell model in the frequency range of 10^−1^–10^5^ Hz and an amplitude of 10 mV. According to the equivalent circuit diagram in Fig. 7(b), the curves after fitting with Z-SimDemo software are displayed in Fig. 7(a) (the inset is an enlarged curves view of f-CuCo_2_S_4_ and n-CuCo_2_S_4_). Obviously, the Nyquist plots of different CEs are composed of two semicircles. Therein, in the semicircle located in the high-frequency region, the starting point of the semicircle is usually defined as the series resistance (*R*_s_), and the radius of the small semicircle represents the charge transfer resistance (*R*_ct_), which can be used to assess the charge transfer process at the CE/electrolyte interface. Besides, the semicircle in the low-frequency region is generated by the diffusion of the electrolyte, which is called the diffusion resistance (*R*_w_). The relevant fitting parameters of various CEs are summarized in Table 1. Generally, the smaller the *R*_s_, the higher the electron transfer efficiency.^40^ The electrochemical performance parameters in Table 1 demonstrate that the *R*_s_ values of f-CuCo_2_S_4_, n-CuCo_2_S_4_, p-CuCo_2_S_4_, and m-CuCo_2_S_4_ CEs are 2.35 Ω, 2.77 Ω, 2.94 Ω, and 3.30 Ω, respectively. Clearly, the f-CuCo_2_S_4_ CE exhibits the lowest *R*_s_ value, which indicates that the f-CuCo_2_S_4_ electrode has the best electron transport performance and electrocatalytic activity. Moreover, among the different CEs, the f-CuCo_2_S_4_ shows the smallest *R*_ct_ value (0.076 Ω). Normally, the *R*_ct_ value is negatively correlated with the electrocatalytic performance of the CEs. The smaller the *R*_ct_, the higher the electrocatalytic activity and conductivity of the CEs. Therefore, the f-CuCo_2_S_4_ electrode presents optimal catalytic performance. Simultaneously, the corresponding Bode curves of different CEs are plotted in Fig. 7(b). The electron lifetime (*τ*_e_) at the CE/electrolyte interface can be obtained by the formula *τ*_e_ = 1/2π*f*_max_, where *τ*_e_ represents the time required for S_n_^2−^ to be reduced to S^2−^, and *f*_max_ is the maximum characteristic peak frequency in the low-frequency region. Apparently, the *τ*_e_ of various CEs can be estimated as f-CuCo_2_S_4_ > n-CuCo_2_S_4_ > p-CuCo_2_S_4_ > m-CuCo_2_S_4_, meaning that the f-CuCo_2_S_4_ CE has the fastest catalytic reduction reaction rate for S_n_^2−^, proving that compared with other CuCo_2_S_4_ CEs, the f-CuCo_2_S_4_ CE has the most outstanding catalytic activity.

**Table 1:** Electrochemical parameters fitting for symmetrical cells fabricated with various CuCo_2_S_4_ CEs

Counter electrode	*R*_s_ (Ω)	*R*_ct_ (Ω)	*R*_w_ (Ω)	*J*_0_ (mA cm^−2^)
f-CuCo_2_S_4_	2.35	0.076	9.78	5.37
n-CuCo_2_S_4_	2.77	0.383	11.36	2.40
p-CuCo_2_S_4_	2.94	0.514	41.42	1.51
m-CuCo_2_S_4_	3.30	0.730	77.87	1.01

Tafel test was used to further investigate the electrocatalytic performance of different CEs. Fig. 7(c) depicts the Tafel polarization curves of CEs with different morphologies, which can reflect two important parameters: the exchange current density (*J*_0_) proportional to the catalytic reaction rate and the limiting current density (*J*_lim_) proportional to the ion diffusion coefficient in the electrolyte. The value of *J*_0_ can be obtained by the intercept of the cathode branch and the zero potential intersection. *J*_lim_ can be attained by the intercept of the vertical axis. The detailed values are summarized in Table 1. Obviously, the values of *J*_0_ (5.37 mA cm^−2^) and *J*_lim_ of the f-CuCo_2_S_4_ CE are greater than those of the CEs with other morphologies, which indicates that the f-CuCo_2_S_4_ CE has higher electrocatalytic activity and faster electrolyte diffusion rate. And the larger the *J*_0_ value, the better the corrosion resistance of the CE in the polysulfide electrolyte. Therefore, from the value of *J*_0_, it can be judged that f-CuCo_2_S_4_ CE has better corrosion resistance than n-CuCo_2_S_4_ (2.40 mA cm^−2^), p-CuCo_2_S_4_ (1.51 mA cm^−2^) and m-CuCo_2_S_4_ (1.51 mA cm^−2^). The above conclusions are consistent with the analysis results of EIS and Bode curves. In addition, to further illustrate the advantages of f-CuCo_2_S_4_ CE, the conductivity (*G*) of different CEs was characterized by linear sweep voltammetry (LSV), and the results are shown in Fig. 7(d). According to the formula *G* = 1/*R* = *I*/*V*, the *G* of each CE can be obtained by fitting the slope of the curve. After calculation, the *G* values of f-CuCo_2_S_4_, n-CuCo_2_S_4_, p-CuCo_2_S_4_ and m-CuCo_2_S_4_ are 0.035 S, 0.021 S, 0.017 S and 0.012 S, respectively. Distinctly, the *G* value of f-CuCo_2_S_4_ CE is significantly higher than that of other CEs, suggesting that the nanoflower-like morphology can more effectively assist the CE to transfer electrons during the operation of QDSSCs, enhance the conductivity of the CE, and thus facilitate the photovoltaic performance of the cell.

**Fig. 8:**
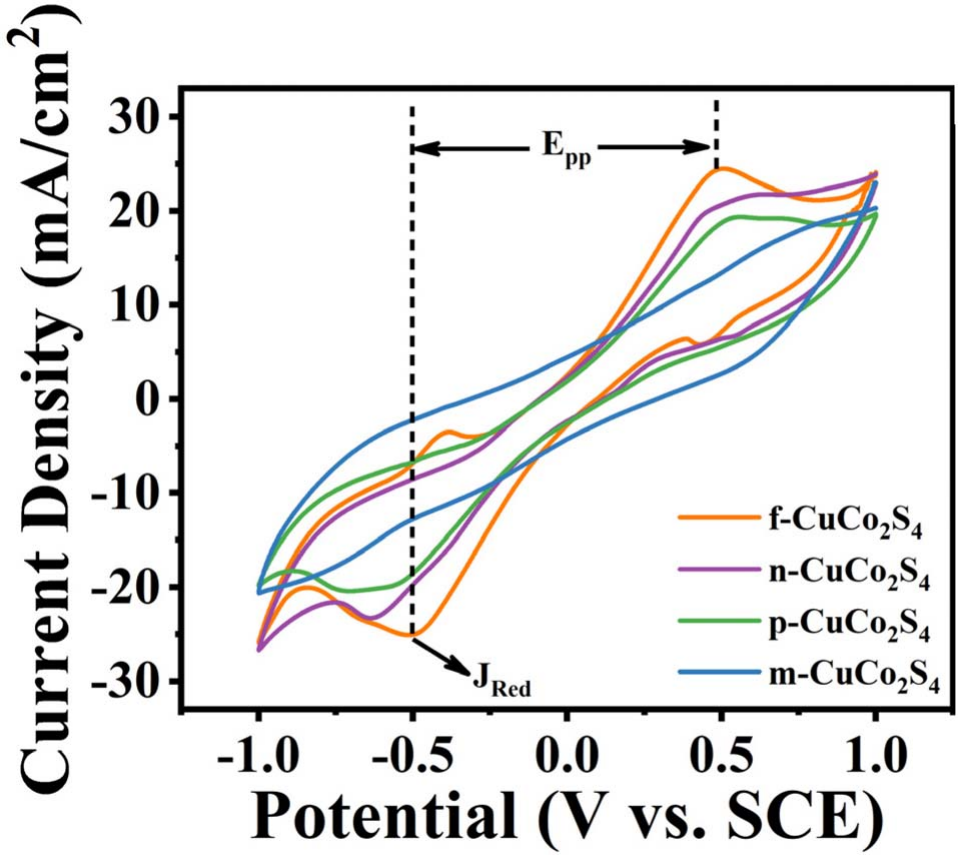
CV curves of different counter electrodes in the three-electrode system.

**Fig. 9:**
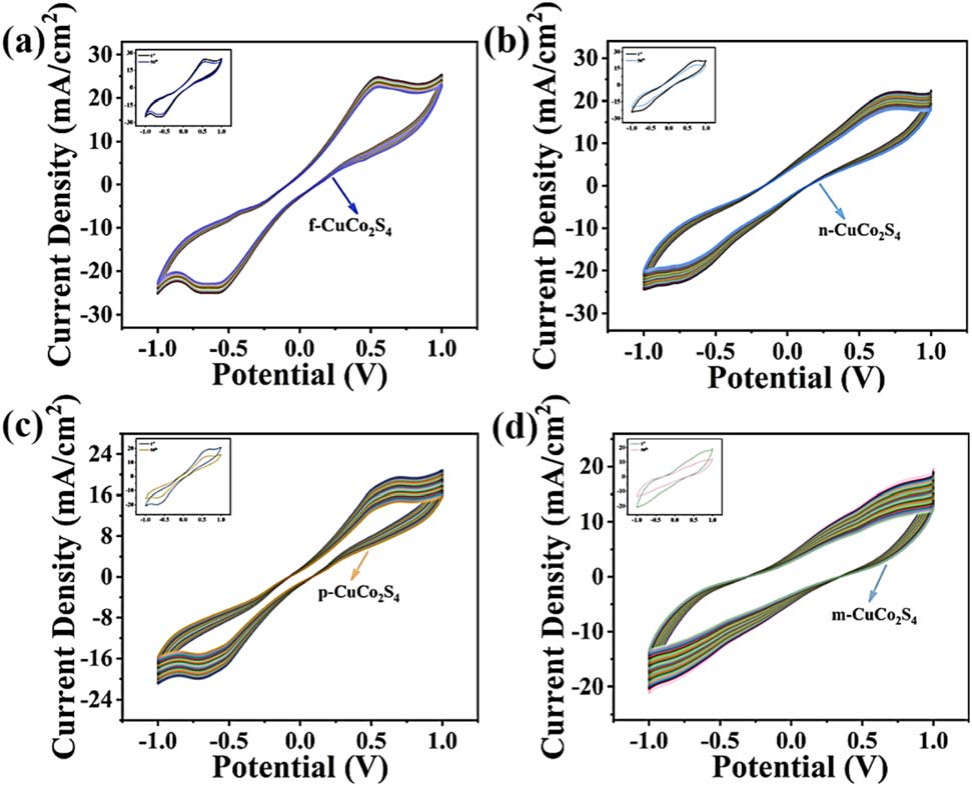
Continuous CV curves of symmetric cell model based on (a) f-CuCo_2_S_4_; (b) n-CuCo_2_S_4_; (c) p-CuCo_2_S_4_; (d) m-CuCo_2_S_4_ counter electrodes.

To better evaluate the ability of the counter electrode to catalyze the reduction of S_n_^2−^ in polysulfide electrolytes, CV tests were performed on different CEs in an electrolyte consisting of 2 M Na_2_S, 2 M sulfur powder and 0.2 M KCl, employing Pt, saturated calomel electrode (SCE) and CuCo_2_S_4_ with different morphologies as the CE, reference electrode and working electrode, respectively. It is generally recognized that a larger reduction peak current density (*J*_Red_) and a smaller peak-to-peak separation (*E*_pp_) contribute to promote the catalytic reduction of CEs. As depicted in Fig. 8, the values of the cathodic peak current density of CuCo_2_S_4_ CEs with different morphologies follow the order of f-CuCo_2_S_4_ > n-CuCo_2_S_4_ > p-CuCo_2_S_4_ > m-CuCo_2_S_4_. Additionally, f-CuCo_2_S_4_ CE exhibits the smallest *E*_pp_ value, indicating that f-CuCo_2_S_4_ CE has better catalytic activity in polysulfide electrolytes than other CEs. Simultaneously, to further study the stability and corrosion resistance of different CEs in polysulfide electrolytes, multi-cycle continuous CV scan tests were carried out using a symmetric cell model. It can be clearly seen from Fig. 9 that after 50 cycles of scanning, f-CuCo_2_S_4_ showed better repeatability compared with n-CuCo_2_S_4_, p-CuCo_2_S_4_ and m-CuCo_2_S_4_ CEs. Besides, f-CuCo_2_S_4_ CE also showed the highest current density value, and its attenuation was negligible. However, the current density of n-CuCo_2_S_4_, p-CuCo_2_S_4_ and m-CuCo_2_S_4_ CEs all revealed a significant downward trend. The above results demonstrate that f-CuCo_2_S_4_ CE has relatively outstanding stability and electrocatalytic activity in polysulfide electrolytes.

**Fig. 10:**
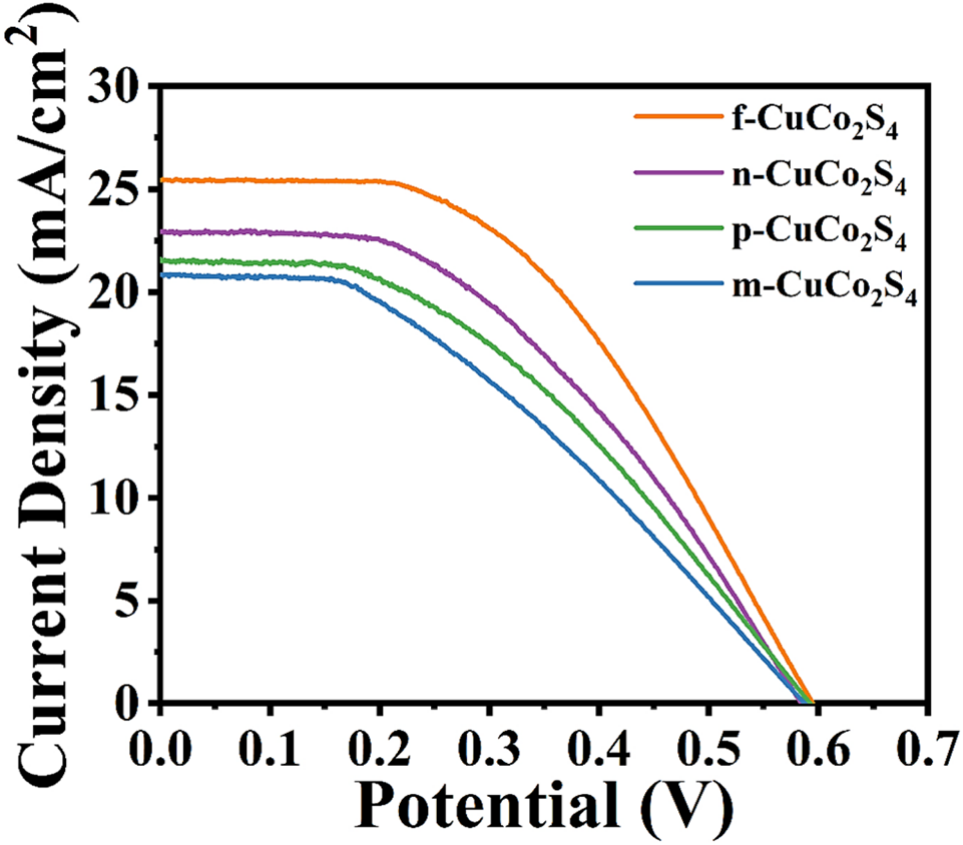
*J*–*V* characteristic curves of QDSSCs based on diverse CEs.

Under AM 1.5G, 100 mW cm^−2^ simulated sunlight, different counter electrodes and FTO/TiO_2_/CdS/CdSe/ZnS photoanodes were assembled into “sandwich” type complete QDSSCs and the photovoltaic performance was characterized. To ensure the stability and repeatability of the experimental data, 10 QDSSCs were used for each test, and the detailed photovoltaic performance parameters (*J*_sc_, *V*_oc_, FF, PCE) obtained are listed in Table 2. Fig. 10 depicts the photocurrent density–voltage (*J*–*V*) curves of QDSSCs equipped with different CEs. As is evident from the figure, the performance parameters of the QDSSC based on f-CuCo_2_S_4_ CE are enhanced (*J*_sc_ = 25.42 mA cm^−2^; *V*_oc_ = 0.596 V; FF = 0.49; PCE = 7.42%), especially the PCE. Compared with QDSSCs assembled with n-CuCo_2_S_4_ (PCE = 5.98%), p-CuCo_2_S_4_ (PCE = 5.33%) and m-CuCo_2_S_4_ CEs (PCE = 4.78%), the PCE of QDSSCs based on f-CuCo_2_S_4_ CE increased by about 24%, 39% and 55%, respectively, which was mainly attributed to the large specific surface area and open three-dimensional porous structure of f-CuCo_2_S_4_, which increased the active sites for catalytic reduction reactions, promoted the effective penetration of electrolytes, and thus accelerated the electron transfer process. Simultaneously, the corresponding error analysis diagram of the PCE of QDSSCs based on four CEs was depicted, and 10 cells were selected as a group for each CE for testing. Fig. S4(a) shows that the average PCE of QDSSCs based on f-CuCo_2_S_4_ CE is 7.40%, demonstrating relatively good reproducibility and stability. For comparison, we also tested the photovoltaic performance of QDSSCs based on conventional Cu_2_S and Pt CEs under the same test conditions. The test results in Fig. S5 and Table S2 show that the PCE of QDSSCs based on f-CuCo_2_S_4_ CE is greatly improved compared with Cu_2_S and Pt CEs. Additionally, the incident photon-to-electron conversion efficiency (IPCE) was used to further test the photovoltaic performance of the cells. As shown in Fig. S6, the QDSSCs have a light absorption range of 400–700 nm, and the QDSSCs based on f-CuCo_2_S_4_-CE exhibited the highest IPCE. Furthermore, the integrated short-circuit current density (*J*_sc_) values calculated from the IPCE are listed in Table S4. Clearly, they agree well with the *J*_sc_ measured from the *J*–*V* curves.

**Table 2:** Detailed photovoltaic parameters of QDSSCs equipped with diverse CuCo_2_S_4_ CEs

Counter electrode	*J*_sc_ (mA cm^−2^)	*V*_oc_ (V)	FF	PCE (%)
f-CuCo_2_S_4_	25.42	0.596	0.49	7.42
n-CuCo_2_S_4_	22.92	0.585	0.45	5.98
p-CuCo_2_S_4_	21.58	0.592	0.42	5.33
m-CuCo_2_S_4_	20.83	0.587	0.32	4.78

**Fig. 11:**
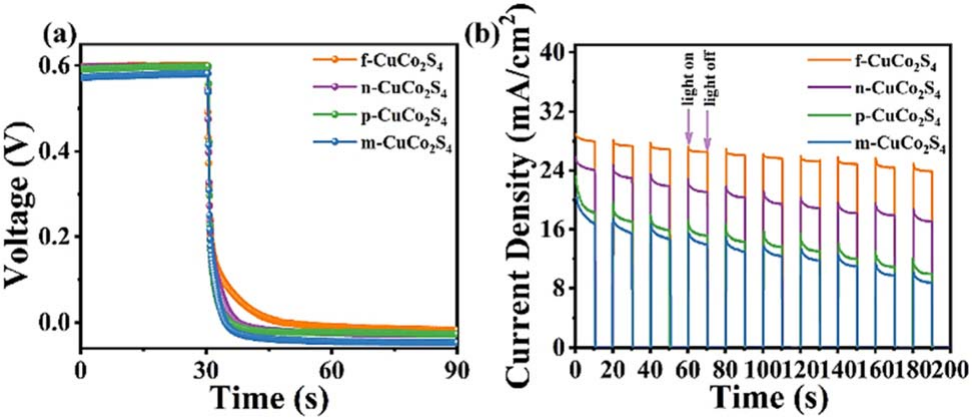
(a) OCVD measurements; (b) on–off tests of QDSSCs based on different CEs.

For further research the photovoltaic performance of QDSSCs based on various CEs, the charge recombination phenomenon at the photoanode/electrolyte interface of different QDSSCs was characterized by open circuit voltage decay (OCVD) test. First, a stable open circuit voltage was obtained under standard sunlight, and then the light source was turned off to further obtain the test curves, which are presented in Fig. 11(a). Researchers generally agree that a slower open circuit voltage decay implies a slower charge recombination rate and a longer electron lifetime. Therefore, it can be seen from Fig. 11(a) that the QDSSC based on f-CuCo_2_S_4_ CE exhibits the slowest decay rate, which indicates that its excellent electrocatalytic activity inhibits the charge recombination at the photoanode/electrolyte interface, thereby improving the conversion efficiency of QDSSCs. Moreover, the startup rate of QDSSCs devices is crucial for the practical application of the device. The light response speed of diverse QDSSCs was studied by on-off experimental measurement, and the results are presented in Fig. 11(b). Obviously, under illumination, the current density of all QDSSCs showed a rapid increase and then stabilized. In the dark state, the current density of the cell displayed a rapid decline. More critically, compared with other QDSSCs, the QDSSCs equipped with f-CuCo_2_S_4_ CE revealed the highest current density, and its current density showed a small attenuation trend after 10 on–off experimental tests. This shows that QDSSCs based on CuCo_2_S_4_ CE have a strong sensitivity to light, and QDSSCs with f-CuCo_2_S_4_ CE have the lowest probability of charge recombination at the photoanode/electrolyte interface.

**Fig. 12:**
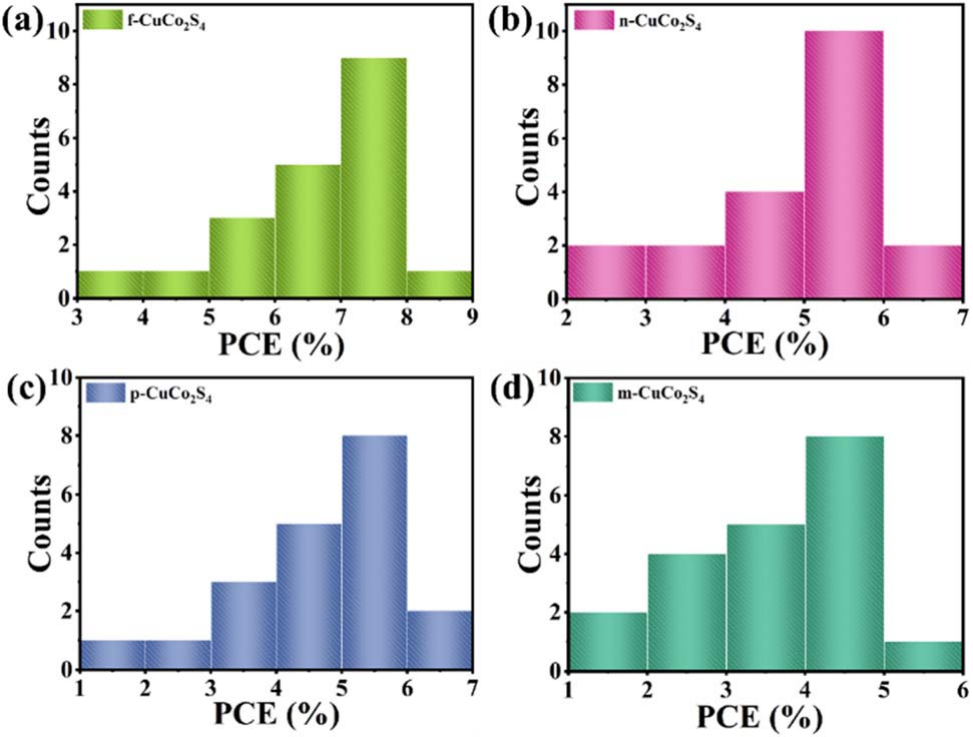
Efficiency statistics histogram of QDSSCs equipped with (a) f-CuCo_2_S_4_; (b) n-CuCo_2_S_4_; (c) p-CuCo_2_S_4_; (d) m-CuCo_2_S_4_ counter electrodes.

Reproducibility tests were conducted to further assess the photovoltaic performance of QDSSCs integrated with distinct CuCo_2_S_4_ CEs under simulated AM 1.5G solar irradiation. Fig. 12(a–d) illustrates the efficiency statistical histograms of QDSSCs based on f-CuCo_2_S_4_, n-CuCo_2_S_4_, p-CuCo_2_S_4_, and m-CuCo_2_S_4_ CEs, respectively, with 20 cells used as samples in each test. Notably, QDSSCs incorporating various CEs exhibit superior reproducibility, and the devices assembled with f- CuCo_2_S_4_ CE show higher PCE. All the above analysis results reveal that the nanoflower-like hierarchical architecture of f-CuCo_2_S_4_ is beneficial to the electrocatalytic activity, and the large specific surface area and open three-dimensional porous structure help to enhance the stability of the electrode, thereby improving the photovoltaic performance and repeatability of QDSSCs.

## Conclusions

CuCo_2_S_4_ with distinct morphologies (f-CuCo_2_S_4_, n-CuCo_2_S_4_, p-CuCo_2_S_4_, m-CuCo_2_S_4_) were prepared by a facile solvothermal method. The preparation method is based on a precursor-directed strategy, which is economical and convenient, avoiding the addition of templates and structure-directing agents. Additionally, this study preliminarily explored the effect of the morphology of CuCo_2_S_4_ CEs on the photovoltaic performance of QDSSCs. The study demonstrated that among diverse CuCo_2_S_4_, nanoflower-like CuCo_2_S_4_ exhibited a three-dimensional porous structure and the largest specific surface area (*S*_BET_ = 44.86 m^2^ g^−1^), which can provide abundant active sites for the catalytic reduction of S_n_^2−^. Concurrently, electrochemical tests clarified that f-CuCo_2_S_4_ CE has the highest electrocatalytic activity and stability in polysulfide electrolytes. Under standard simulated solar illumination (AM 1.5G, 100 mW cm^−2^), the f-CuCo_2_S_4_ CE-assembled QDSSCs can achieve a PCE of 7.42%, accompanied by *J*_sc_ = 25.42 mA cm^−2^, *V*_oc_ = 0.596 V, and FF = 0.49, which are 24%, 39% and 55% higher than those of QDSSCs based on n-CuCo_2_S_4_ (PCE = 5.98%), p-CuCo_2_S_4_ (PCE = 5.33%) and m-CuCo_2_S_4_ (PCE = 4.78%) CEs, respectively, and are about 40% higher than the highest conversion efficiency (5.28%) of existing CdS/CdSe/ZnS-sensitized QDSSCs equipped with a CuCo_2_S_4_ CE. This study provides ideas for the preparation of CuCo_2_S_4_-based CE materials with different morphologies by precursor-guided strategy, and realizes the coordinated optimization of morphology control, electrochemical and photovoltaic performance, providing a new pathway for the further development of high-performance CuCo_2_S_4_-based composite counter electrode materials.

## Author contributions

Qiu Zhang: conceptualization, resources, investigation, visualization, writing – original draft, writing – review & editing. Yuekun Zhang: software, formal analysis, writing – review & editing. Xuemei Fu: data curation, software. Prof. Chunxiao Zhang and Prof. Xiuyan Jiang: supervision.

## Conflicts of interest

There are no conflicts to declare.

## Supplementary Material

RA-015-D5RA06421J-s001

## Data Availability

The data that supports the findings of this study is available from the corresponding authors upon reasonable request. Supplementary information is available. See DOI: https://doi.org/10.1039/d5ra06421j.

## References

[cit1] Chaudhary N., Jain A., Pahuja M., Das S., Jyoti J., Harini E. M., Rani S., Siddiqui S. A., Rani D., Afshan M., Sharangi S., Bera C., Ghosh K. (2025). Sol. RRL.

[cit2] Panchenko V. A., Kovalev A. A., Chakraborty S. (2025). Int. J. Hydrogen Energy.

[cit3] Kazemian A., Xiang C. (2025). Renew. Sustain. Energy Rev..

[cit4] Zhu Y., Lan J., Du Y., Yang T., Yu H., Liang T., Lv X., Cheng C., Ji J. (2025). Chem. Eng. J..

[cit5] Mahmoud S. A., Mansour A. F., Elsisi M. E. (2025). Sci. Rep..

[cit6] Peng Y., Zhou R., Wang L., Gao Y., Li X., Yang X., Lü W. (2025). J. Mater. Chem. A.

[cit7] Tartuci L. G., Raphael E., Carvalho Junior J. A., Machado W. S., Schiavon M. A. (2025). Energy Technol..

[cit8] Zhang L., Zhang T., Cui D., Wang C., Yu H., Li F. (2025). J. Power Sources.

[cit9] Dang H. P., Tran L., Bao L. H., Le H. N. T. (2025). RSC Adv..

[cit10] Zhang Q., Zhang Y., Zhang T., Li F., Xu L. (2023). J. Alloys Compd..

[cit11] Zhang Q., Zhang Y., Li F., Zhang T., Xu L. (2023). J. Alloys Compd..

[cit12] Li Q., Zhang T., Cui D., Li F. (2024). Dalton Trans..

[cit13] Li Q., Zhang T., Cui D., Xu L., Li F. (2024). Dalton Trans..

[cit14] Wang S., Zhou Y., Huang B., Qi J., Hua M., Jin X., Li L. (2025). Surf. Interfaces.

[cit15] Zhang Q., Zhang T., Wang L., Li F., Xu L. (2022). Dalton Trans..

[cit16] Kasaye B. B., Shura M. W., Dibaba S. T. (2025). Micro Nanostruct..

[cit17] Zhang Q., Jin L., Zhang Y., Zhang T., Li F., Xu L. (2021). Dalton Trans..

[cit18] Zhang Q., Jin Z., Li F., Xia Z., Yang Y., Xu L. (2020). Sol. Energ. Mat. Sol. C..

[cit19] Zhang T., Zhang Q., Wang Y., Wang L., Li F., Xu L. (2022). J. Alloys Compd..

[cit20] Abinaya S., Sakthivel R., Parthibavarman M., Al-Mohaimeed A. M., Al-onazi W. A., Alam M. W. (2024). J. Mater. Sci.: Mater. Electron..

[cit21] Zhang T., Yu H., Zhang L., Yang X., Wang C., Li F. (2025). ACS Appl. Nano Mater..

[cit22] Liu S. A., Wang S., Cao Y., Liang C., Geng S., Guo H., Liu Y., Zhang W., Li L. (2022). Sol. Energy.

[cit23] Abuali M., Arsalani N., Ahadzadeh I. (2022). Electrochim. Acta.

[cit24] Vanasundari K., Mahalakshmi G., Ravichandran K. (2024). J. Mater. Sci.: Mater. Electron..

[cit25] Shombe G. B., Razzaque S., Khan M. D., Nyokong T., Mashazi P., Choi J., Bhoyate S., Gupta R. K., Revaprasadu N. (2021). RSC Adv..

[cit26] Zhao Y., Luo Y., Sun B., Li T., Han S., Dong Z., Lin H. (2022). Compos. B: Eng..

[cit27] Huang J., Zhu Y., Zhong H., Yang X., Li C. (2014). ACS Appl. Mater. Interfaces.

[cit28] Naveenkumar P., Maniyazagan M., Kang N., Yang H. W., Kim S. J. (2025). Electrochim. Acta.

[cit29] Sun W., Ling X., Wei W., Hu H., Jiang Z., Yan Z., Xie J. (2019). Appl. Surf. Sci..

[cit30] Alizadeh A., Roudgar-Amoli M., Shariatinia Z., Abedini E., Asghar S., Imani S. (2023). Renew. Sustain. Energy Rev..

[cit31] Sarilmaz A., Ozen A., Akyildiz H., Siyahjani Gultekin S., Kus M., Ozel F. (2021). Sol. Energy.

[cit32] Siddiq M., Saxena S., Al Souwaileh A., Subbiah J., Anandan S. (2024). Opt. Mater..

[cit33] Baptayev B., Mustazheb D., Abilova Z., Balanay M. P. (2020). Chem. Commun..

[cit34] Zhang T., Zhang Q., Li Q., Li F., Xu L. (2023). Chem. Eng. J..

[cit35] Wan Khalit W. N. A., Mustafa M. N., Sulaiman Y. (2019). Results Phys..

[cit36] Li B., Yuan F., He G., Han X., Wang X., Qin J., Guo Z. X., Lu X., Wang Q., Parkin I. P., Wu C. (2017). Adv. Funct. Mater..

[cit37] Moosavifard S. E., Fani S., Rahmanian M. (2016). Chem. Commun..

[cit38] Yang Z., Zhang S., Fu Y., Zheng X., Zheng J. (2017). Electrochim. Acta.

[cit39] You H., Zhang L., Jiang Y., Shao T., Li M., Gong J. (2018). J. Mater. Chem. A.

[cit40] Zhang T., Zhang Q., Wang Y., Li F., Xu L. (2021). Dalton Trans..

