# Supplementary material for: Morphology-controlled synthesis of CuCo2S4 as a high-efficiency counter electrode via a precursor-directed strategy for quantum dot-sensitized solar cells (QDSSCs)
Source: RSC Adv. 2025 Oct 27;15(48):40666–74. doi: 10.1039/d5ra06421j (PMC12557471; doi:10.1039/d5ra06421j)
Supplement: RA-015-D5RA06421J-s001 [file RA-015-D5RA06421J-s001.pdf]

Supplementary Information (SI) for RSC Advances.
This journal is © The Royal Society of Chemistry 2025

Supporting Information

Morphology-Controlled Synthesis of $CuCo_2S_4$ as a High-Efficiency Counter Electrode via Precursor-Directed Strategy for Quantum Dot-Sensitized Solar Cells (QDSSCs)

Qiu Zhang [a, b, c*], Yuekun Zhang [a, b], Chunxiao Zhang [a], Xiuyan Jiang [a], Xuemei Fu [a]

a. School of Chemical Engineering, Shandong Institute of Petroleum and Chemical Technology, Dongying 257061, China.

b. Shandong Key Laboratory of Green Electricity&Hydrogen Science and Technology, Shandong Institute of Petroleum and Chemical Technology, Dongying, 257061, China.

c. Dongying Key Laboratory of New Energy Materials and Devices, School of Chemical Engineering, Shandong Institute of Petroleum and Chemical Technology, Dongying, 257061, China.

[*] Corresponding authors Tel.: +86 546 7396190; Fax: +86 546 7396190.

E-mail addresses: 2023042@sdipct.edu.cn

1. Experimental section

1.1. Chemicals and materials

$CuCl_2·2H_2O$ (AR, ≥99.0%), $CoCl_2·6H_2O$ (AR, ≥99.0%), $Cu(NO_3)_2·3H_2O$ (AR, ≥99.5%), Terpineol ($C_{10}H_{18}O$, AR), H_2NCSNH_2 (AR, ≥99.0%), $CuSO_4·5H_2O$ (AR, ≥98.0%), $CoSO_4·7H_2O$ (AR, ≥98.0%), $Cu(CO_2CH_3)_2·xH_2O$ (AR, ≥98.0%), $Co(CO_2CH_3)_2·4H_2O$ (AR, ≥98.0%), $C_4H_6O_4Zn·2H_2O$ (AR, ≥99.0%), $Cd(NO_3)_2·4H_2O$ (AR, ≥98.0%), KCl (AR, ≥99.5%), Na_2SO_3 (AR, ≥97.0%), Ethylene glycol, ethanol, absolute methanol and acetone were purchased from Sinopharm. Ethyl cellulose (CP), $Na_2S·9H_2O$ (AR, ≥98.0%), $Co(NO_3)_2·6H_2O$ (AR, ≥99%), $CdSO_4·8/3H_2O$ (AR, 99.0%), Sulfur (S, 99.99%), Titanium oxide (TiO_2, Degussa, P25), $N(CH_2COONa)_3$ (AR, 98.0%), selenium powder (Se, 200 mesh, 99.9%) were purchased from Aladdin (Sigma-Aldrich).

1.2. Synthesis of $CuCo_2S_4$ with other morphologies

The nanosheet-like (n-$CuCo_2S_4$) was prepared by a simple solvothermal reaction method. Briefly, 0.5 mmol of $Cu(NO_3)_2·3H_2O$ was added to 60 mL of ethylene glycol solution and stirred until it was dissolved. Then, 1 mmol of $Co(NO_3)_2·6H_2O$ and 3 mmol of thiourea were added to the above solution successively. After continuous stirring for 30 min, the mixed solution was transferred to a 100 mL autoclave and reacted at 200 °C for 12 h, and the heating rate was 2 °C min^{-1}. After the reaction cooled down to room temperature, the obtained precipitate was washed three times with deionized water and ethanol respectively. Then, it was transferred into an oven at 60 °C and dried for 10 h, thus the n-$CuCo_2S_4$ could be obtained. The synthesis method of particle-like (p-$CuCo_2S_4$) requires replacing the precursor salt with $CuSO_4·5H_2O$ and $CoSO_4·7H_2O$, while the precursor salt of particle-like (p-$CuCo_2S_4$) is

$Cu(CO_2CH_3)_2 \cdot xH_2O$ and $Co(CO_2CH_3)_2 \cdot 4H_2O$. The rest of the preparation steps are the same as those of n-$CuCo_2S_4$.

1.3. Synthesis of Na_2SeSO_3

Na_2SeSO_3 was prepared by blending appropriate concentration of selenium powder and Na_2SO_3 refluxing at 80°C for 4 h.

1.4. Characterizations

X-ray powder diffraction test was conducted from 10 to 80° adopting Siemens D5005 diffractometer with Cu target Kα (λ = 1.5418 Å) rays as X-ray source. X-ray photoelectron spectroscopy (XPS) was carried out applying an ESCALABMKII spectrometer and the X-ray source was achromatic Al-Ka (1486.6 eV). A field emission scanning electron microscope (SEM JEOL JSM 4800F) was used to study the surface morphology. The electron transmission microscopy (TEM), HRTEM images and element composition of the samples was received using the transmission electron microscope JEOL-2100F equipped with X-ray energy dispersion spectrometer (EDS) analysis. The data of nitrogen adsorption–desorption isotherms were collected from an ASAP 2020 (Micromeritics, USA). The EIS, Tafel, CV and LSV all used CHI760E electrochemical workstation (Shanghai Chenhua, China). EIS test conditions: the frequency range is 10^{-1}-10^{5} Hz; the amplitude is 0.01 V, which is performed under the condition of open-circuit voltage. All characterizations were conducted at ambient temperature and pressure. An IVIUM purchased from Tianjin Brillante Technology Limited with a filtered 500 W Xenon lamp is utilized to conduct on-off tests, current–voltage (I–V) curves and open circuit voltage decay (OCVD) measurements under the condition of AM 1.5 G 100 mW cm^{-2}.

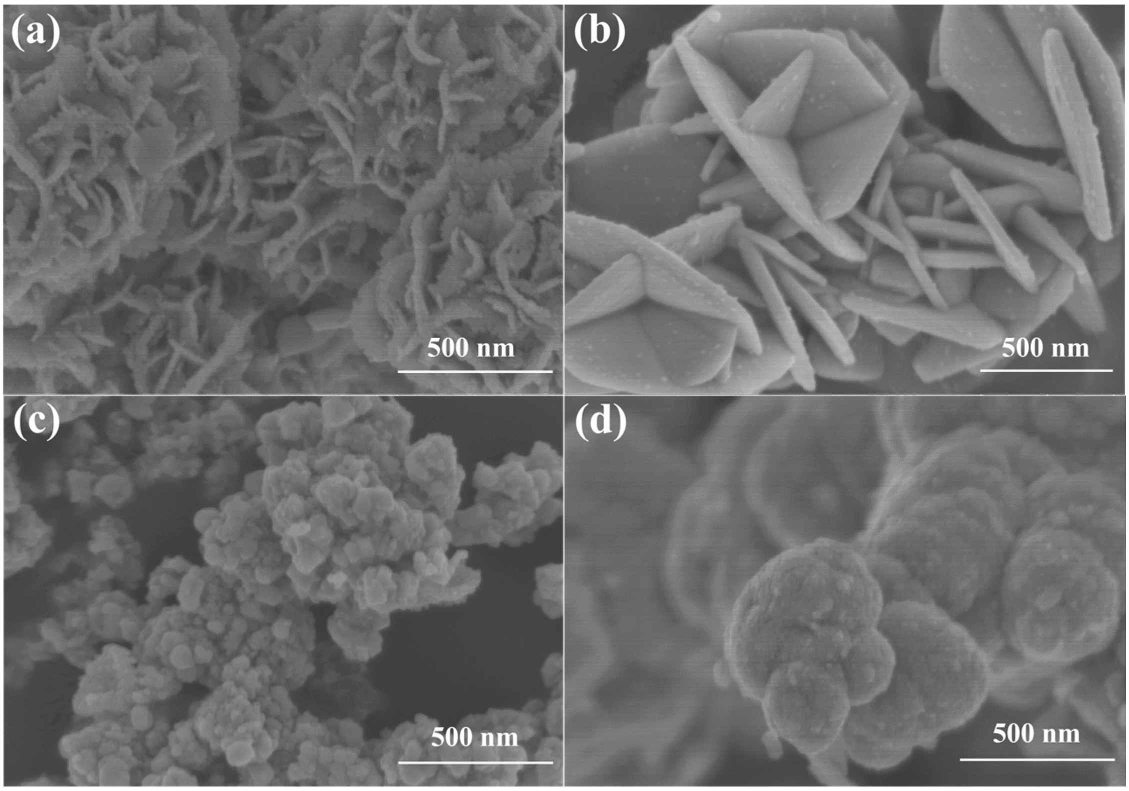

Fig. S1 SEM images of (a) flower-like; (b) nanosheet-like; (c) nanoparticle-like; (d) microsphere-like $CuCo_2S_4$.

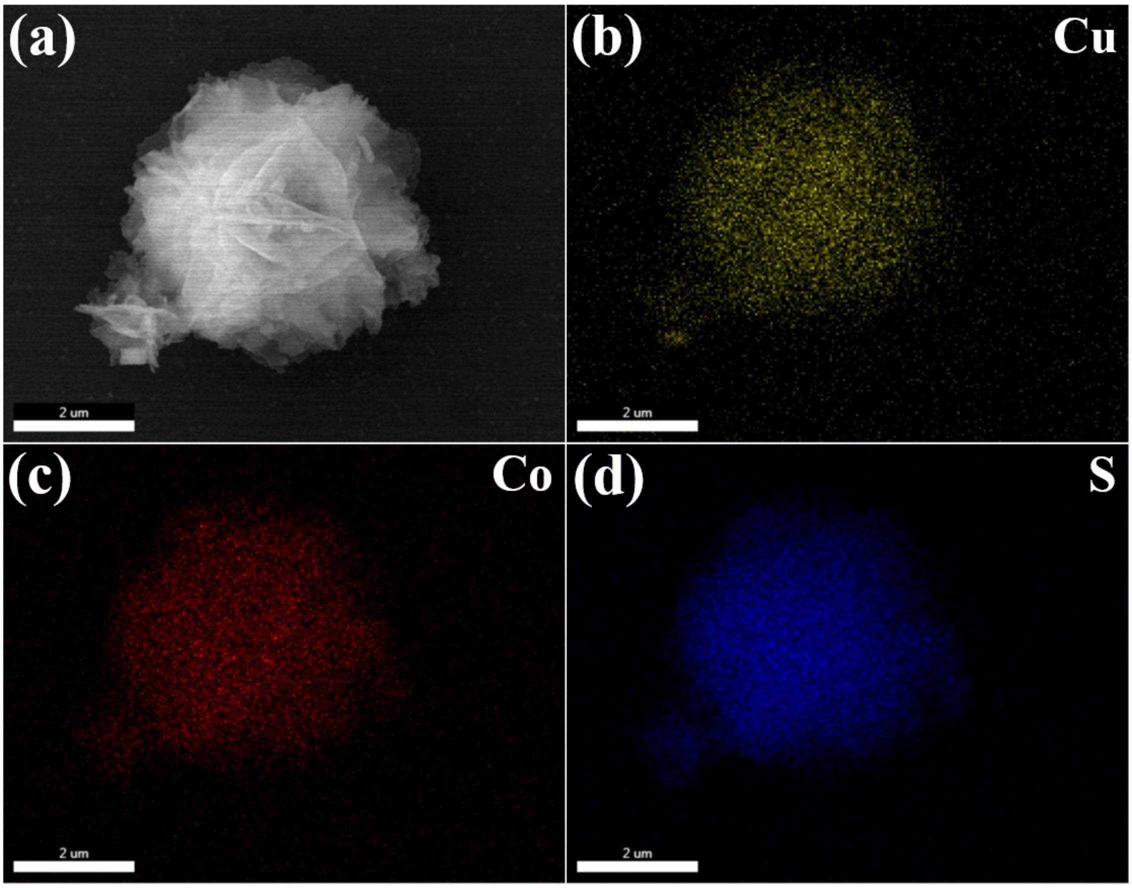

Fig. S2 The SEM EDS element distribution images in flower-like $CuCo_2S_4$.

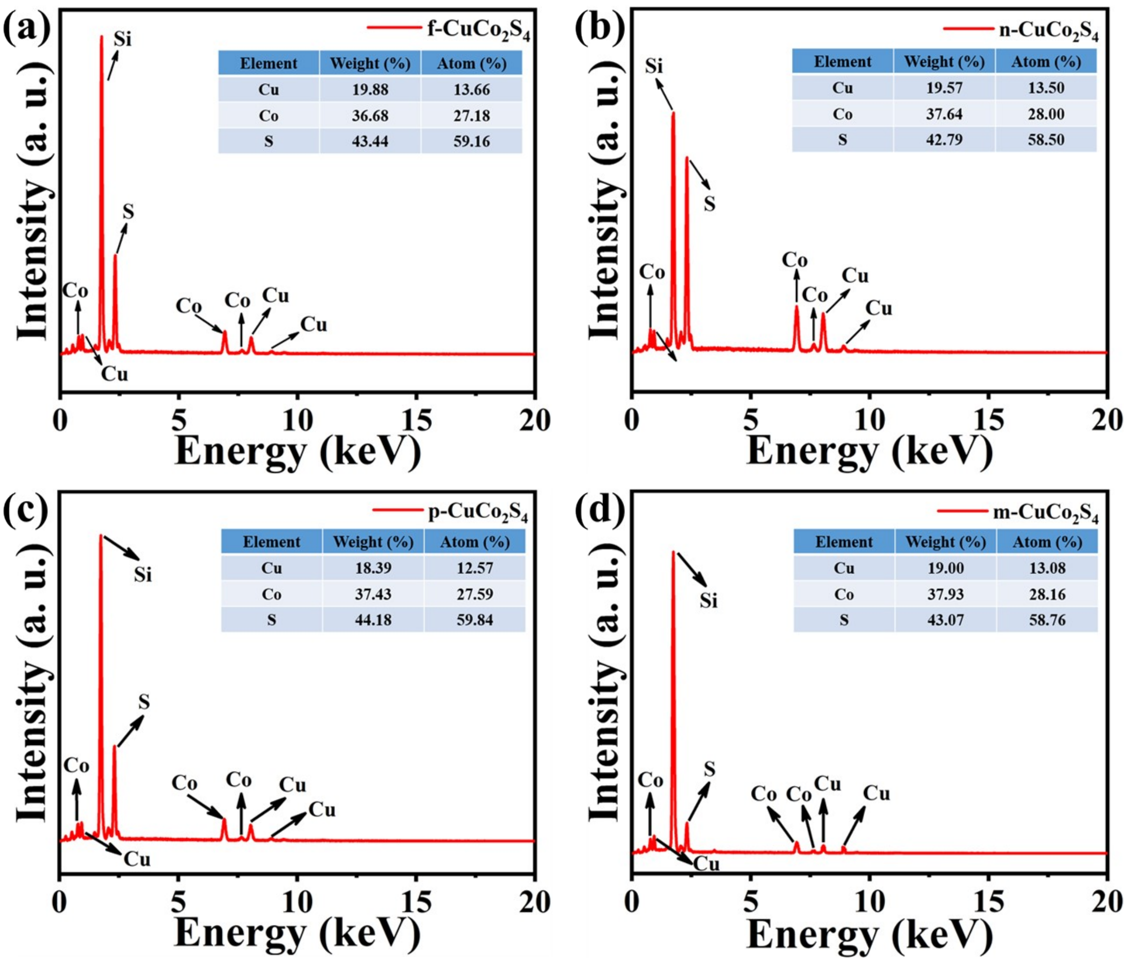

Fig. S3 EDS spectrum of (a) f-$CuCo_2S_4$, (b) n-$CuCo_2S_4$, (c) p-$CuCo_2S_4$, and (d) m-$CuCo_2S_4$.

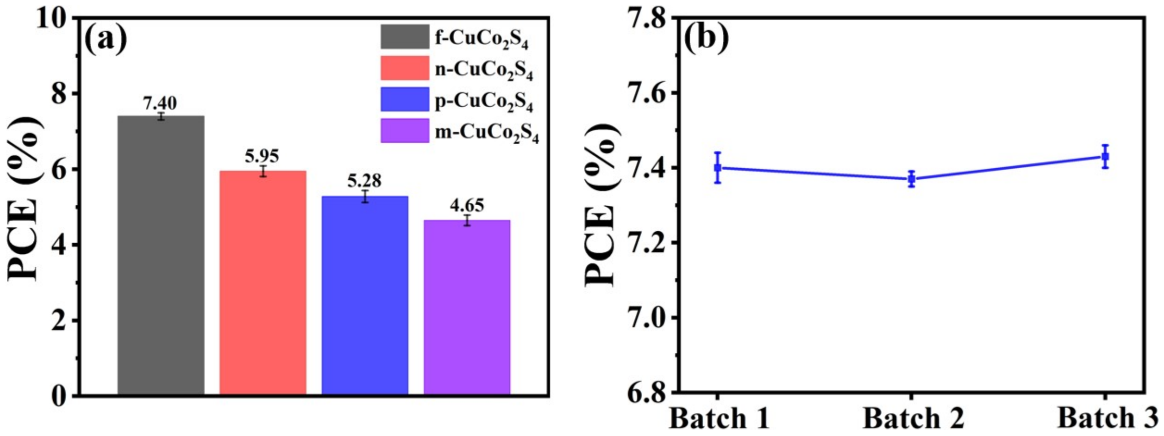

Fig. S4 (a) Comparison of average PCEs of f-$CuCo_2S_4$, n-$CuCo_2S_4$, p-$CuCo_2S_4$, m-

$CuCo_2S_4$ CEs fabricated QDSSCs; (b) Comparison of the average PCEs of 3 batches of QDSSCs based on f-$CuCo_2S_4$ CEs.

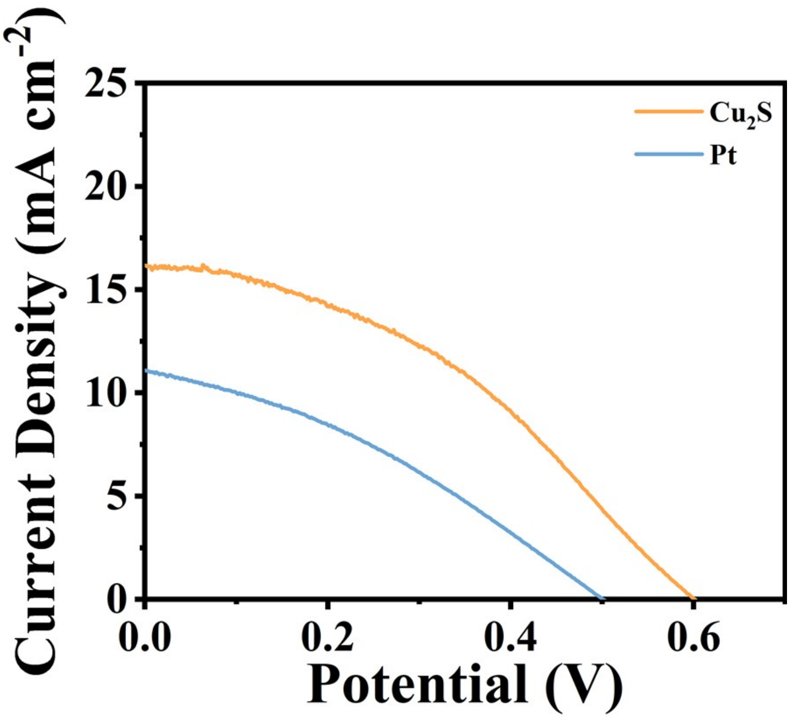

Fig. S5 (a) J-V characteristic curves of QDSSCs based on Cu_2S and Pt CEs.

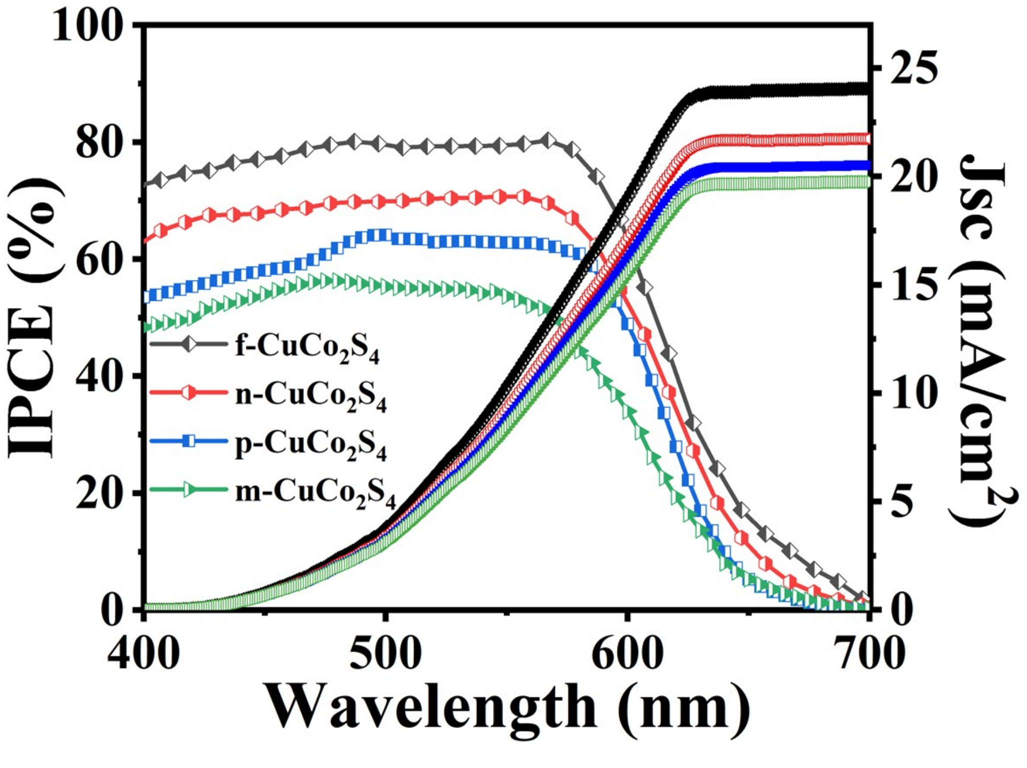

Fig. S6 IPCE of QDSSCs with diferent counter electrodes.

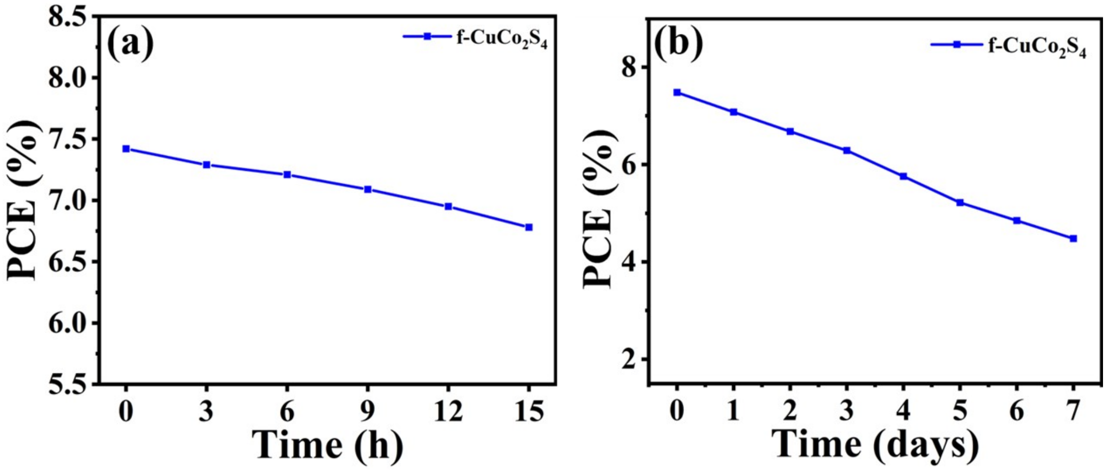

Fig. S7 (a) Continuous illumination; (b) Multi-day stability test of QDSSCs based on f-$CuCo_2S_4$ CEs.

Table S1

Summary of detailed BET data for the different $CuCo_2S_4$ materials.

**Sample**	S_{BET} **(m^2 g^{-1})**
**f-$CuCo_2S_4$**	44.86
**n-$CuCo_2S_4$**	14.52
**p-$CuCo_2S_4$**	35.43
**m-$CuCo_2S_4$**	30.62

Table S2

Detailed photovoltaic parameters of QDSSCs equipped with Cu_2S and Pt CEs.

**Counter electrode**	J_{sc} **(mA cm^{-2})**	V_{oc} **(V)**	**FF**	**PCE (%)**

**Cu_2S**	16.17	0.60	0.38	3.75
**Pt**	11.09	0.50	0.33	1.83

Table S3

Photovoltaic performance parameters of QDSSCs based on other reported metal sulfide counter electrodes.

Counter electrodes	Photoande	J_{sc} (mA cm^{-2})	V_{oc} (V)	FF	PCE (%)	Reference
$CuCo_2S_4$	TiO_2/CdS/CdSe/ZnS	22.10	0.575	0.520	6.59	[1]
$NiCo_2S_4$	TiO_2/CdSe/ZnS	15.58	0.550	0.489	4.22	[2]
CoS	TiO_2/CdS/CdSe/ZnS	17.19	0.651	0.516	5.77	[3]
$Cu_{0.5}Ni_{0.5}Co_2S_4$	TiO_2/CdS/CdSe/ZnS	18.11	0.590	0.49	5.20	[4]
Cu_2SnS_3	ZnO/ZnSe/CdSe	11.46	0.810	0.437	4.06	[5]
Cu_7S_4/Co_9S_8	TiO_2/Mn-CdS/CdSe/ZnS	23.42	0.672	0.540	8.43	[6]
Cu_2WS_4	TiO_2/CdS/CdSe/ZnS	22.75	0.600	0.430	5.92	[7]
CuS	TiO_2/CdS/CdSe/ZnS	14.31	0.603	0.490	4.27	[8]
Cu_2S/PbS	TiO_2/CdS/CdSe/ZnS	18.08	0.550	0.536	5.28	[9]
1T-MoS_2	TiO_2/CdS/CdSe/ZnS	15.03	0.586	0.440	3.92	[10]
Cu_2S	TiO_2/CdS/CdSe/ZnS	16.20	0.560	0.414	3.77	[11]
Cu_7S_4	TiO_2/CdS/CdSe/ZnS	19.09	0.480	0.494	4.53	[12]
PbS	TiO_2/CdS/CdSe/ZnS	11.20	0.560	0.550	3.48	[13]

FeS_2	ZnO/ZnSe/CdSe/ZnS	13.58	0.743	0.387	3.90	[14]
(CdCuCoMnZn)S_x	TiO_2/Mn-CdS/CdSe/ZnS	25.60	0.665	0.49	8.33	[15]
f-$CuCo_2S_4$	TiO_2/CdS/CdSe/ZnS	25.42	0.596	0.49	7.42	**This work**

[1] T. Zhang, Q. Zhang, Y. Wang, L. Wang, F. Li, L. Xu, $CuCo_2S_4$ hollow spherical shell supported by RGO as an efficient counter electrode for QDSSCs: Shortening electron migration paths and promoting electron migration, J. Alloys Compd. 2022, 911, 164981.

[2] J. Xiao, X. Zeng, W. Chen, F. Xiao, S. Wang, High electrocatalytic activity of self-standing hollow $NiCo_2S_4$ single crystalline nanorod arrays towards sulfide redox shuttles in quantum dot-sensitized solar cells, Chem. Commun. 2023, 49, 11734-11736.

[3] X. Chen, Y. Zhuang, Q. Shen, X. Cao, W. Yang, P. Yang, In situ synthesis of $Ti_3C_2T_x$MXene/CoS nanocomposite as high performance counter electrode materials for quantum dot-sensitized solar cells, Sol. Energy. 2021, 226, 236-244.

[4] Z. Liu, Z. Tian, Q. Chen, Q. Zhong, Improving the electrocatalytic activity and stability of spinel sulfide counter electrodes by trimetallic synergy effects for quantum dot sensitized solar cells, New J. Chem. 2021, 45, 4766-4772.

[5] J. Xu, X. Yang, T. L.Wong, C. S. Lee, Large-scale synthesis of Cu_2SnS_3 and $Cu_{1.8}S$ hierarchical microspheres as efficient counter electrode materials for quantum dot sensitized solar cells, Nanoscale. 2012, 4, 6537-6542.

[6] Q. Zhang, Y. Zhang, T. Zhang, F. Li, L. Xu, Efficient and stable heterostructured Co_9S_8/Cu_7S_4 composite counter electrodes derived from Prussian blue analogs for quantum dot-sensitized solar cells, J. Alloy. Compd. 2023, 930, 167455.

[7] Q. Zhang, Y. Zhang, T. Zhang, F. Li, L. Xu, Polyoxometalate-modified ternary copper-tungsten-sulfide nanocrystals as high-performance counter electrode materials for quantum dotssensitized solar cells, J. Alloy. Compd. 2023, 938, 168633.

[8] C. D. Sunesh, C. V. V. M. Gopi, M. P. A. Muthalif, H. Kim, Y. Choe, Improving the efficiency of quantum-dot-sensitized solar cells by optimizing the growth time of the CuS counter electrode, Appl. Surf. Sci. 2017, 416, 446-453.

[9] I. R. Jo, J. A. Rajesh, Y. H. Lee, J. H. Park, K. S. Ahn, Enhanced electrocatalytic activity and electrochemical stability of Cu_2S/PbS counter electrode for quantum-dot-sensitized solar cells, Appl. Surf. Sci. 2020, 525, 146643.

[10] Z. Tian, Q. Chen, Q. Zhong, Honeycomb spherical 1T-MoS_2 as efficient counter electrodes for quantum dot sensitized solar cells, Chem. Eng. J. 2020, 396, 125374.

[11] A. K. Prasad, I.-R. Jo, S.-H. Kang, K.-S. Ahn, Novel method for synthesis of reduced graphene oxide–Cu_2S and its application as a counter electrode in quantum-dot-sensitized solar cells, Appl. Surf. Sci. 2021, 564, 150393.

[12] M. Wang, W. Chen, J. Zai, S. Huang, Q. He, W. Zhang, Q. Qiao, X. Qian, Hierarchical Cu_7S_4 nanotubes assembled by hexagonal nanoplates with high catalytic performance for quantum dot-sensitized solar cells, J. Power Sources. 2015, 299, 212-220.

[13] C. V. Thulasi-Varma, S. S. Rao, K. D. Ikkurthi, S.-K. Kim, T.-S. Kang, H.-J.

Kim, Enhanced photovoltaic performance and morphological control of the PbS counter electrode grown on functionalized self-assembled nanocrystals for quantum-dot sensitized solar cells via cost-effective chemical bath deposition, J. Mater. Chem. C. 2015, 3, 10195-10206.

[14] J. Xu, H. Xue, X. Yang, H. Wei, W. Li, Z. Li, W. Zhang, C.-S. Lee, Synthesis of honeycomb-like mesoporous pyrite FeS_2 microspheres as efficient counter electrode in quantum dots sensitized solar cells, Small. 2014, 10, 4754-4759.

[15] T. Zhang, H. Yu, L. Zhang, X. Yang, C. Wang, F. Li, High-Entropy Metal Sulfides $(CdCuCoMnZn)S_x$ as High-Performance Counter Electrode for Quantum Dot-Sensitized Solar Cells, ACS Appl. Nano Mater., 2025, 8, 1160-1172.

Table S4

The J_{sc} parameters obtained from the IPCE and J-V curves of QDSSCs with different counter electrodes.

**Counter electrode**	**Integrating current from IPCE (mA/cm^2)**	**J_{sc} from J-V curves (mA/cm^2)**
**f-$CuCo_2S_4$**	24.06	25.42
**n-$CuCo_2S_4$**	21.75	22.92
**p-$CuCo_2S_4$**	20.50	21.58
**m-$CuCo_2S_4$**	19.76	20.83
